# The Gut Microbiota-Bile Acids-TGR5 Axis Mediates *Eucommia ulmoides* Leaf Extract Alleviation of Injury to Colonic Epithelium Integrity

**DOI:** 10.3389/fmicb.2021.727681

**Published:** 2021-08-18

**Authors:** Zhenya Zhai, Kai-Min Niu, Yichun Liu, Chong Lin, Xin Wu

**Affiliations:** ^1^Jiangxi Functional Feed Additive Engineering Laboratory, Institute of Biological Resource, Jiangxi Academy of Sciences, Nanchang, China; ^2^CAS Key Laboratory of Agro-ecological Processes in Subtropical Region, Institute of Subtropical Agriculture, Chinese Academy of Sciences, Changsha, China; ^3^National Engineering Laboratory for Pollution Control and Waste Utilization in Livestock and Poultry Production, Changsha, China; ^4^College of Animal Science and Technology, Jiangxi Agricultural University, Nanchang, China; ^5^Tianjin Institute of Industrial Biotechnology, Chinese Academy of Sciences, Tianjin, China

**Keywords:** *Eucommia ulmoides* leaves extracts, inflammatory bowel disease, gut microbiota, bile acids, epithelial barrier, Caco-2

## Abstract

*Eucommia ulmoides* leaves (EL) are rich in phenolic acids and flavonoids, showing enhancing intestinal health effects. The intestinal microbiota-bile acid axis plays important roles in the occurrence and recovery of inflammatory bowel disease (IBD). However, whether EL extract (ELE) has regulatory effects on the intestinal microbiota, bile acid metabolism, and IBD is still unclear. To fill this gap, 2% dextran sulfate sodium (DSS)-induced mild IBD in a C57BL/6J mouse model that was treated with 200 or 400 mg/kg (intake dose/body weight) ELE was used. Oral ELE supplementation alleviated DSS-induced shortening of colon and colonic epithelial injury. Compared with the DSS group, ELE supplementation significantly decreased Toll-like receptor 4 (TLR4) and interlukin-6 (IL-6) and increased occludin and claudin-1 mRNA expression level in the colon (*p* < 0.05). Combined 16S rRNA gene sequencing and targeted metabolomic analyses demonstrated that ELE significantly improved the diversity and richness of the intestinal microbiota, decreased the abundance of *Bacteroidaceae*, and increased *Akkermansiaceae* and *Ruminococcaceae* abundance (*p* < 0.05) compared with DSS-induced IBD mice. Moreover, ELE significantly increased the serum contents of deoxycholic acid (DCA) and tauroursodeoxycholic acid (TUDCA), which were highly positively correlated with *Akkermansia* and unidentified_*Ruminococccaceae* relative to the DSS group. We then found that ELE increased Takeda G-protein coupled receptor 5 (TGR5), claudin-1, and occludin mRNA expression levels in the colon. In the Caco-2 cell model, we confirmed that activation of TGR5 improved the reduction in transepithelial electoral resistance (TEER) and decreased the permeability of FITC-dextran on monolayer cells induced by LPS (*p* < 0.05). siRNA interference assays showed that the decrease in TGR5 expression led to the decrease in TEER, an increase in FITC-dextran permeability, and a decrease in claudin-1 protein expression in Caco-2 cells. In summary, ELE alleviated IBD by influencing the intestinal microbiota structure and composition of bile acids, which in turn activated the colonic *TGR5* gene expression in the colon and promoted the expression of tight junction proteins. These findings provide new insight for using ELE as a functional food with adjuvant therapeutic effects in IBD.

## Introduction

In Western countries, the incidence rate of inflammatory bowel disease (IBD) exceeds 0.3% (more than 2 million people), while in some Asian countries (such as China and Japan), the incidence of IBD also has begun to increase rapidly (Ng et al., 2017). IBD can aggravate inflammation, and then induce a lower level of tight junction protein expression between gut epithelial cells, resulting in structural damage to the gut epithelium, decrease of transepithelial electrical resistance (TEER), an increase in macromolecular permeability, and invasion of harmful pathogenic microorganisms (Citi, 2018; Mehandru and Colombel, 2021). The main factor that induces IBD is the disorder of intestinal microbiota and host metabolism caused by high-fat and high-protein “Western diet” (Tang et al., 2019; Sinha et al., 2020). Therefore, an effective way to prevent and improve IBD is adjusting the diet and increasing the intake of functional food, to improve the intestinal microbiota community and host metabolism.

Appropriate bacterial community structure and homeostasis are essential for gastrointestinal health. Studies have shown that increase in *Bacteroides*, *Proteobacteria*, and *Enterobacteriaceae* have been found in the intestinal contents and feces of IBD mice and human patients (Zhai Z. et al., 2019). Probiotics such as *Akkermansia* and *Lactobacillus* were enriched in mice at the convalescent stage of IBD. Additionally, probiotic intervention or fecal bacteria transplantation has been effective in relieving IBD symptoms (Lee and Chang, 2020).

Metagenomic and metabolomic studies have shown that metabolic changes in response to the intestinal microbiota may be the inducement or protective factor of IBD (Lee and Chang, 2020). Bile acids (BAs), short-chain fatty acids (SCFAs), and tryptophan metabolites play essential roles in gut epithelial repair, homeostasis, and immune regulation (Lee and Chang, 2020). Bile acids, a class of microbiota-host-related metabolites, play a role in regulating gut inflammation, barrier function, and cell proliferation (Connors et al., 2020; Sinha et al., 2020; Sorrentino et al., 2020). Secondary bile acids such as Lithocholic acid (LCA) and deoxycholic acid (DCA) can alleviate dextran sulfate sodium (DSS)-induced IBD by promoting intestinal stem cell proliferation and generation of regulatory T cells (Campbell et al., 2020; Sorrentino et al., 2020). In addition, partially hydrophilic bile acids such as tauroursodeoxycholic acid (TUDCA) and ursodeoxycholic acid (UDCA), showed beneficial effect on DSS-induced IBD in mice through reducing endoplasmic reticulum stress (Cao et al., 2013) and intestinal barrier disruption (Wang W. et al., 2018). The BA receptor TGR5-related pathway may play a key role in epithelium barrier function and formation (Xu et al., 2015; Merlen et al., 2020; Sinha et al., 2020). In biliary epithelium, activation of TGR5 enhanced the barrier function and decreased epithelium permeability (Merlen et al., 2020). In the intestinal crypt, TGR5 was only expressed in Lgr5-labeled intestinal stem cells and was necessary for DSS-induced recovery of IBD in mice (Sorrentino et al., 2020). However, whether TGR5 can regulate colon epithelium barrier function remains unclear.

*Eucommia ulmoides* tea, is a well-known Chinese functional food made from *Eucommia ulmoides* leaves. In recent years, EL extract (ELE) has been found to have beneficial effects on immune regulation, antioxidants, bone injury wound healing, and intestinal health enhancement (Zhu and Sun, 2018). EL extract, for example, has been reported to alleviate intestinal epithelium injury, which induced by obesity in mice (Murakami et al., 2018). Although the potential mechanism of ELE in alleviating IBD remains unclear, the protective effect may be attributed to bioactive components such as polyphenols, flavonoids, and polysaccharides in ELE. The flavonoids within ELE can relieve intestinal oxidative stress in piglets (Xiao et al., 2019). Polyphenols from blueberries have been shown to expand *Akkermansia* and *Bifidobacterium*, increase LCA and chenodeoxycholic acid (CDCA), and decrease hyodeoxycholic acid (HDCA) (Guo et al., 2019). Polysaccharides are the carbon source of gut microbiota and promote the proliferation of *Bifidobacterium* and *Lactobacillus*, reduce the colonization of pathogenic bacteria (Schroeder et al., 2018). Taken together, ELE exhibits antioxidant, anti-inflammatory, and immunomodulatory functions. However, whether ELE has a regulating effect on IBD alleviation, gut microbiota, and bile acid metabolism is still unclear.

To clarify this situation, with the purpose of evaluating the potential regulatory effects of compounds in ELE on IBD, microbiota, and bile acids, we employed a 2% DSS-induced IBD mouse model combined with ELE intervention. The gut microbiota and serum bile acid composition were also then investigated by integrated microbiome-metabolomic methods. Furthermore, using a Caco-2 cell model, we evaluated the repair effect of bile acids on lipopolysaccharide-induced monolayer epithelial barrier injury and its potential mechanism.

## Materials and Methods

### Ethics Statement

In this study, the experimental design and procedures in this study were reviewed and approved by the Committee of the Institute of Subtropical Agriculture at the Chinese Academy of Sciences. All the experiment operations were conducted following the guidelines of the institute of Subtropical Agriculture on Animal Care, Chinese Academy of Sciences (No. ISA-2020-18).

### Preparation of Plant Extracts

*Eucommia ulmoides* leaves (EL) were grinded and filtered with 2 mm screener. The EL powder (500 g) was extracted by soaking twice in 5 L 95% alcohol for 7 days at room temperature, and the collected supernatant was filtered and concentrated using vacuum evaporator. Subsequently, the EL concentrate was decolorized with petroleum ether and then rotated again, then the extract of ELE was obtained by vacuum freeze-drying.

### Determination of Total Flavonoids, Total Phenolic Acid, and Sugars in ELE

The content of total flavonoid and total phenolic acids in ELE was determined by using the plant flavonoids test kit (A142-1-1, Nanjing Jiancheng Bioengineering Institute, Nanjing, China) and plant total phenols test kit (A143-1-1, Nanjing Jiancheng Bioengineering Institute). The content of sugar was determined by using the plant total sugar and reducing sugar test kit (Shanghai Yuanye Bio-Technology Co., Ltd, Shanghai). All operations are in accordance with the relevant instructions. The content of total flavonoid, total phenolic acids, total soluble sugar, and reducing sugar in ELE was 44.59, 89.27, 104.23, and 25.81 mg/g, respectively.

### Small Molecular Metabolites in ELE Detected by LC-MS/MS Analysis

The small molecule metabolites in ELE were detected by using liquid chromatography tandem secondary mass spectrometry (LC-MS/MS). The instrument parameters are as follows: LC was conducted by using UltiMate 3000 (Thermofisher, Waltham, MA, United States) and equipped with C18 chromatographic column (the column temperature is 18°C during operation). Mobile phase was A: 100% acetonitrile + 0.1% formic acid, B: H_2_O + 0.1% formic acid. Elution gradient: A: 0−7 min, 5−50%; 7−8 min, 50−75%; 8−9 min, 75−80%; 9−11 min, 80−90%; 11−15 min, 90−95%; 15−20 min, 95%. Velocity of flow: 0.2 ml/min. MS/MS was conducted by using Q-Exactive Focus (Thermofisher, Waltham, MA, United States). Ion source: ESI source, atomization temperature: 300°C, atomization gas (sheath gas) pressure: 40 ARB, auxiliary gas pressure: 10 ARB; transmission capillary temperature: 3p 20°C, scanning mode: (1) full scan, resolution 35,000, in source CID: 0 ev; ddms2, resolution 17,500, HCD-stepped NCE 10, 30, and 50. All the reagents for LC-MS were purchased from Sigma-Aldrich; the mass spectrum data were compared with the mzVault and MassList database. The relative content of metabolites was reflected by peak area. The relative content of metabolites was reflected by peak area. The relative abundance of the small molecular components is presented in peak area relative to the total peak area. Geniposide and chlorogenic acid accounted for 12% and 10%, respectively. The top 30 small molecular metabolites are shown in Supplementary Table 1.

### Establishment of 2% DSS-Induced IBD Model in Mice and Treated With Different Dose of ELE

C57BL/6 mice (11 weeks of age) were purchased from STJ Laboratory Animal Co., LTD (Hunan, China). The mice were housed in four per cage under the same condition (temperature, 25 ± 1°C; lighting cycle, 12 h:12 h light/dark; 8:00−20:00 for light) and had free access to food and drinking water. Dextran sulfate sodium (36,000–5,000 MW) was purchased from MP Biomedicals (Santa Ana, CA, United States) and dissolved in drinking water to 2% (w/v) and given *ad libitum* to mice beginning on day 0 for 7 days (Tanaka et al., 2003). EL extract were dissolved in distilled water. The mice were then gavage with distilled water (CON and DSS group), 200 and 400 mg/kg (according to the body weight) ELE for 11 days (D5–D8). All the mice were evaluated in terms of body weight change and diarrhea index every day.

### Sample Collection

At the end of this experiment, all the mice were fasted for 6 h, and anesthesia was induced by intraperitoneal injection of 2% pentobarbital sodium (45 mg/kg body weight). The blood samples were collected in 1.5 ml centrifuge tubes (germ-RNase- and DNase-free) after enucleation of the eyeball. The distal ileum (near the cecum to 2 cm) and colon were collected and stored in 4% neutral polyformaldehyde fixative or frozen in liquid nitrogen for real-time quantitative PCR (RT-qPCR) or western blotting. The cecum contents were collected in sterile enzyme free centrifuge tube and frozen in liquid nitrogen, and then stored at −80°C until total genomic DNA extraction. The genomic DNA was used for subsequent 16S rRNA gene sequencing analysis.

### Colon Tissue Histological Examination

The colonic tissue was embedded in paraffin, cut into 5 μm sections, and stained with hematoxylin and eosin (H&E). The colon crypt depth was observed by microscope (Olympus, Japan), and the depth of crypt was measured using VistarImage (Olympus, Japan) software, matching with the Olympus microscope (Zhai Z. et al., 2019).

### RNA Extraction and Real-Time Quantitative PCR

To evaluate the inflammatory and colon epithelium integrity, we assessed the expression level of genes related to inflammation, tight junction, and bile acid receptor. RNA was extracted from colon tissue using column RNA extraction kit (R4121, Magen, Guangzhou, China). The total RNA concentration was measured using a NanoDrop 2000C spectrophotometer (Thermo Fisher Scientific, Waltham, MA, United States). The total RNA was reverse transcribed into cDNA by using cDNA synthesis kit (CW2582M, CWBIO, Jiangsu, China). The RT-qPCR primers were designed and synthesized by a commercial service of Sangon Biotech (Shanghai, China); the primer sequences are shown in Supplementary Table 2. The RT-qPCR was then performed according to the direction of FastSYBR Mixtrue (CW0955M, CWBIO) and run in the ABI 7500 FAST system (Applied Biosystems Instruments, Thermo Fisher Scientific, United States). The values of the target genes were normalized by the housekeeping gene.

### 16S rRNA Gene Sequencing With Ion S5^TM^ XL

The process of genomic DNA extraction, quality detection and sequencing operation of intestinal microbiota were performed by the methods described in our previous study (Zhai Z. et al., 2019). Briefly, the sequencing raw data was obtained the Ion S5^TM^ XL platform. The raw data was measured by Cutadapt (version 1.9.1) to obtain the clean data. Operational taxonomy units (OTUs) were clustered with 97% identity by using Uparse (v7.0.1001). The non-metric multidimensional scaling (NMDS) were conducted based on OTU by the using R software. OTU is annotated and divided into phylum, class, order, family, genus, and species. The linear discriminant analysis (LDA) effect size (LEfSe) was used to elucidate the differences among bacterial taxa. An LDA score ≥4 was considered to be an important contributor to the model. The Spearman’s analysis was used to measure the correlation between gut microbiota and bile acids.

### Serum Bile Acid Analysis

The BA concentration in serum was measured by LC-MS. The standard curves of 25 BAs were established (Supplementary Table 3). Internal standards (Supplementary Table 4) were added into 50 μl serum samples and concentrated in a concentrator. After concentration, samples were redissolved with 100 μl 50% methanol-H_2_O solution (v:v). LC was conducted by using LC30AD (Shimadzu, Japan) and equipped with C18 chromatographic column. Mobile phase was composed of solution A: acetonitrile (containing 0.01% acetic acid) and solution B: H_2_O (0.01% acetic acid and 5 mmol/L ammonium acetate). Elution gradient is shown in Supplementary Table 5. MS was conducted by using QTRAP 6500 (ABSCIEX, MA, United States). Ion source: ESI source, scanning mode: negative ion scanning, Sperry voltage: 4,500 V (negative), capillary temperature: 550°C, curtain gas: 35 psi. The content of bile acid in blood was calculated by standard curve.

### Preparation and Culture of Caco-2 Cell

Human colon cell line Caco-2 was kindly provided by Professor Baichuan Deng (College of Animal Science, South China agricultural University). The cell was cultured in DMEM/F12 (1:1) medium supplemented with 10% fetal bovine serum (FBS) (Gibco, Thermo Fisher, Waltham, MA, United States) and then incubated at 37°C in a humidified atmosphere containing 5% CO_2_ in air. For transwell experiment, 1 × 10^6^ cells were seeded in 0.32 cm^2^ (0.4 μm) transwell plate and detect the TEER until differentiation is complete (Xiaojun et al., 2020). The cells were then treated with 40 μg/ml LPS (derived from *Escherichia coli*, O111: B4, Beyotime Biotechnology, Shanghai, China) for and treated with 12.5 μM TGR5 agonist INT-777 (I884046, ≥ 99%, MedChemExpress, Shanghai, China) at the same time, then the TEER was detected at 24h and 48h. For TGR5 RNA interference assay, the operation was carried out according to the kit instructions. In brief, negative control (NC) and TGR5-siRNA were mixed with serum-free DMEM/F12 medium and Lipo8000^TM^ (Beyotime Biotechnology) and incubated at room temperature for 20 min. The mix was then added to each well and treated for 24 and 48 h. The NC and TGR5-siRNA sequences were designed and synthesized by (Sangon Biotech) and the sequence were shown in Supplementary Table 6. The FITC-dextran (M.w.10000, MACKLIN, Shanghai, China) permeability of monolayer cell detection has been described in a previous study (Zhai et al., 2018). Briefly, 200 μl FITC-dextran solution (dissolved in HBSS, 5 mg/ml) was added to the apical compartment of transwell plate and 500 μl HBSS were added to basal compartment and cultured for 2 h. The absorbance in the basal compartment was measured using microplate reader (Spark10M, TECAN, Männedorf, Switzerland), and the content of FITC-dextran was calculated relative to a standard curve.

### Cell Protein Sample Collection and Western Blot Analysis

For the experiment, 1 × 10^6^ cells were seed in cell culture dish (6 cm diameter, JET, Guangzhou, China). After 24 h of being seeded, the cells were treated with LPS, LPS + INT-777, and INT-777 for 24 h. Caco-2 samples were lysed in Radio Immunoprecipitation Assay Lysis buffer (RIPA) lysate buffer (containing 1 mM PMSF) on ice for 15 min and then collected. RIPA buffer and PMSF are purchased from Beyotime Technology (Shanghai, China). The total protein concentration was detected by using BCA protein assay kit (Pierce BCA Protein Assay Kit, Beyotime technology) and mixed with loading buffer, and the protein was denatured in a metal bath (95°C, 5 min). In electrophoresis, 60 μg protein samples were loaded in each sampling well. After electrophoresis and membrane transfer, the primary and secondary antibodies were incubated, and electrochemiluminescence (ECL) was detected. The primary and secondary antibodies were shown as follows: claudin-1 (51-9000) was purchased from Thermo Fisher Scientific. TGR5 (NBP2-23669) were purchased from NOVUS (CO, United States). Goat antimouse (bs-40296G-HRP) and goat anti-rabbit (bs-0295G-HRP) antibodies were purchased from Bioss (Beijing, China). In order to detect proteins with similar molecular weight, we used striping buffer to remove antibodies, and then performed blocking, first antibody and second antibody incubation, and ECL chemiluminescence detection. The gray value of protein bands was measured by ImageJ (National Institutes of Health, Germany). The relative protein expression was expressed by the ratio of target protein to β-actin.

### Statistical Analysis

Data are expressed as the mean ± SEM. One-way ANOVA and the least significance difference (LSD) method were used to determine the differences among the groups by using SPSS 20.0 (IBM, SPSS, United States) with significant criteria set to ^∗^*p* < 0.05, ^∗∗^*p* < 0.01, and ^∗∗∗^*p* < 0.001. GraphPad Prism 7 (GraphPad Software Inc., San Diego, CA, United States) was used to generate statistical plots.

## Results

### The Therapeutic Effect of ELE on DSS-Induced IBD in Mice

**FIGURE 1:**
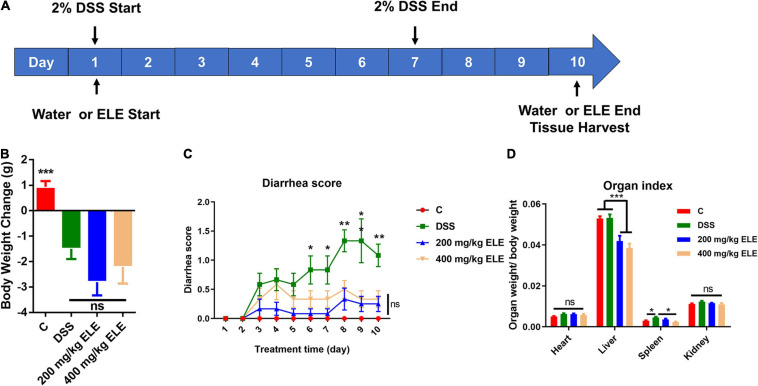
Effects of ELE on body weight, diarrhea score, and organ index in mice. **(A)** The experimental process of this study. **(B)** Oral administration of ELE could not improve DSS-induced weight loss in mice. **(C)** Oral administration of ELE significantly improved DSS-induced diarrhea in mice. **(D)** Oral administration of ELE significantly decreased liver index and improved spleen index induced by DSS. Differences in data in mouse subjects were assessed by one-way ANOVA. *n* = 10–12 mice/group. *p < 0.05; **p < 0.01; ***p < 0.001. ELE, *Eucommia ulmoides* leaf extract; DSS, dextran sulfate sodium.

To evaluate the therapeutic effect of ELE on IBD, we constructed IBD mouse model using 2% DSS in drinking water for 1 week and administered different doses of ELE by gavage for 10 days, as shown in Figure 1A. Compared with the DSS group, ELE did not promote the recovery of body weight loss (Figure 1B) but remarkably attenuated the diarrhea status (Figure 1C, *p* < 0.05). In addition, oral administration alleviated DSS-induced splenic hypertrophy (Figure 1D).

### The Effect of ELE on Colon Length, Epithelium Morphology, and the mRNA Expression of Inflammatory Factors

**FIGURE 2:**
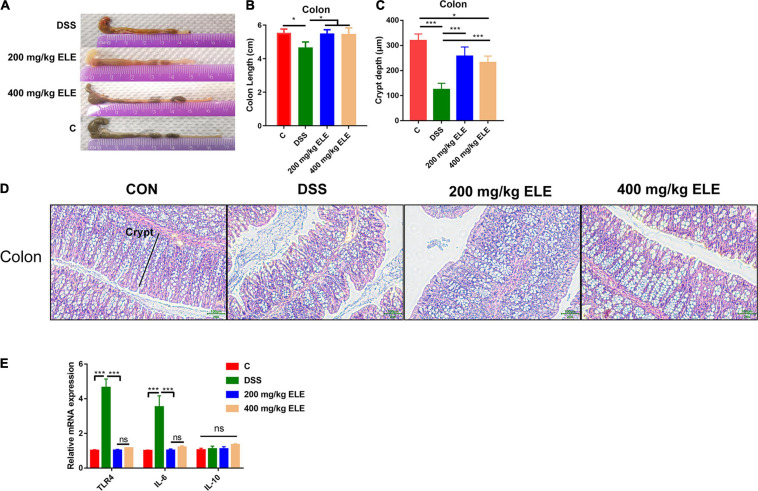
Oral administration of ELE improved DSS induced colon length shortening and epithelial destruction. **(A)** Representative picture of colon length. **(B)** Colon length of mice in each group, *n* = 10–12 mice/per group. **(C)** The crypt depth of mice in colon, *n* = 6 mice/group. **(D)** Representative picture of colon epithelium, objective × 20, the scale indicates 100 μm. **(E)** The relative mRNA expression level of Toll-like receptor 4 (TLR4), IL-6, and IL-10 in colon, *n* = 9–12 mice/group. Differences in data in mouse subjects were assessed by one-way ANOVA. *p < 0.05; ***p < 0.001.

The effects of oral ELE administration on colon length and the epithelium morphology were evaluated. ELE supplementation alleviated the DSS-induced shortening of colon length (*p* < 0.05, Figures 2A,B) and crypt depth (*p* < 0.05, Figures 2C,D). Furthermore, ELE administration downregulated inflammation-related gene expression in the colon, including TLR4 and IL-6, which were more highly expressed in the DSS-treated group (Figure 2E).

### The Effect of ELE on the Gut Microbiota in DSS-Treated IBD Mice

**FIGURE 3:**
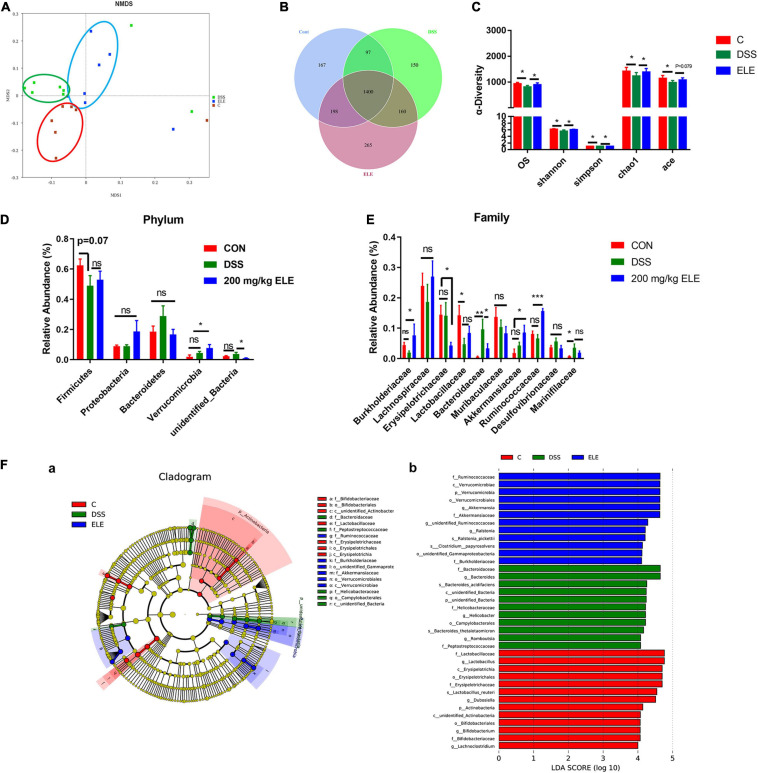
Effects of ELE on the gut microbiota in mice. **(A)** The NMDS plot of gut microbiota. **(B)** The Venn plot based on OTUs of gut microbiota. **(C)** The OS, diversity, and abundance index of gut microbiota. **(D)** Oral administration of ELE regulates the microbiota of IBD mice in phylum level. **(E)** Oral administration of ELE regulates the microbiota of IBD mice in family level. **(F)** Identification of characteristic microbiota in cecal contents of mice with different treatments by LEfSe analysis, **(a)** LefSe taxonomic cladogram and **(b)** LDA score. *n* = 7–8 mice/group. Differences in data in mouse subjects were assessed by one-way ANOVA. *p < 0.05; **p < 0.01; ***p < 0.001. NMDS, non-metric multidimensional scaling. OTU, operational taxonomic unit; LEfse, LDA effect size; LDA, linear discriminant analysis.

Oral administration of 200 and 400 mg/kg ELE showed similar effects in the preliminary results, thus 200 mg/kg was chosen for assessment in the following experiments. The supplementary effect of 200 mg/kg ELE on the gut microbiota in DSS-induced mice was further investigated. The rank abundance and rarefaction curve showed that the selected sequences were sufficient to determine most bacterial diversity (Supplementary Figure 1). The NMDS plot showed a clear separation of the gut microbiota among the control, ELE, and DSS groups (Figure 3A). The Venn plot showed that there were 1,862, 2,023, and 1,807 OTUs in control, ELE, and DSS groups, respectively (Figure 3B). The observed species (OS) and Shannon, Simpson, Chao1, and ACE indexes showed that ELE supplementation recovered the DSS-induced decline in OS and Chao1 index-dependent richness and Simpson and Shannon index-dependent diversity of the microbiota (Figure 3C). The phyla *Firmicutes*, *Proteobacteria*, *Bacteroidetes*, *Verrucomicrobia*, and *unidentified_Bacteria* were the microbiota with the top 5 relative abundances (Figure 3D). Compared with the DSS group, ELE increased the relative abundance of *Verrucomicrobia*. At the family level, DSS significantly reduced the relative abundance of *Lactobacillaceae* and increased the levels of *Bacteroides* and *Marinifilaceae* compared with the control group. After ELE treatment, the relative abundances of *Burkhoderiaceae*, *Akkermansiaceae*, and *Ruminococcaceae* were increased, while those of *Erysipelotrichaceae* and *Bacteroidaceae* were decreased compared with those in the DSS group (Figure 3E). LEfSe analysis demonstrated that *Bacteroidetes*, *Marinifilaceae*, and *Helicobacteraceae* were the main features in IBD mice. *Akkermansia* belonging to the *Verrucomicrobia* phylum was a feature in ELE-administrated mice. However, compared with the control group, the relative abundances of *Lactobacillus*, *Dubosiella*, and *unidentified_Ruminococcaceae* belonging to *Firmicutes* were significantly decreased in IBD- and ELE-treated mice (Figure 3F).

### Effect of ELE on Bile Acid Composition in Serum of Mice

**FIGURE 4:**
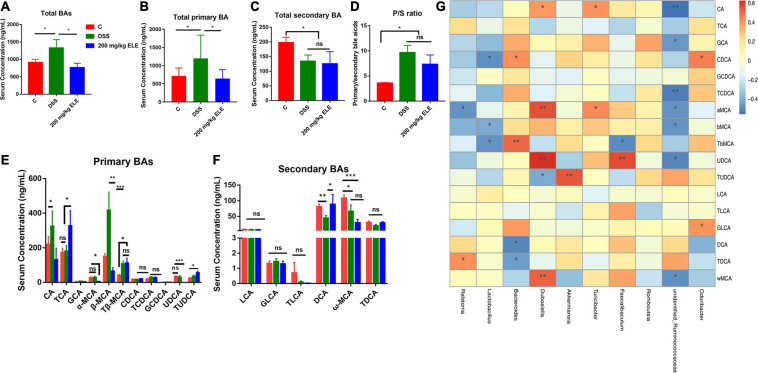
Oral administration of ELE regulated the bile acids through gut microbiota regulatory effect in mice. **(A)** Content of total BA in serum. **(B)** The content of total primary BA in serum. **(C)** Content of total secondary BA in serum. **(D)** Ratio of primary bile acid to secondary bile acid content in serum. **(E)** Qualitative and quantitative analysis of primary bile acids. **(F)** Qualitative and quantitative analysis of secondary bile acids. **(G)** Correlation analysis of gut microbiota and bile acids. *p < 0.05; **p < 0.01; ***p < 0.001.

The content of bile acids in the serum of mice was analyzed by LC-MS. ELE administration reduced the DSS-induced changes higher in total bile acids (*p* < 0.05) and tended to reduce the DSS-induced changes higher in total primary bile acids (0.05 < *p* < 0.1) in the serum (Figures 4A,B). Compared with the control group, ELE administration did not recover the DSS-induced changes of total secondary bile acids (Figure 4C) or the P/S ratio (Figure 4D).

Furthermore, we analyzed the composition of bile acids in the serum of mice in each group. Compared with the control group mice, DSS significantly increased the content of CA and β-MCA and decreased the DCA and ω-MCA content (*p* < 0.05, Figures 4E,F). EL extract treatment restored CA, β-MCA, and DCA to normal levels. Additionally, compared with the DSS and control groups, ELE significantly increased the contents of TCA and TUDCA (*p* < 0.05). The intestinal microbiota can regulate the process of bile acid synthesis, uncoupling, and secondary bile acid production in mice. We analyzed the correlation between bile acid content and the relative abundance of intestinal microbiota (Figure 4G). Consistent with the above results, in normal mice, *unidentified_Ruminococcaceae*, *Lactobacillus* showed a negative correlation with primary bile acids, such as β-MCA, Tβ-MCA TCDCA, CA (−0.55 < R^2^ < −0.40). Bacteroides showed positive correlation with primary bile acids such as Tβ-MCA and CDCA (0.4 < R^2^ < 0.5),while with negative correlation with DCA and TDCA (0.4 < R^2^ < 0.5). In addition, *Dubosiella* showed strong positive correlation with α-MCA, UDCA and ω-MCA (*R*^2^ = 0.54, 0.62, and 0.51). The feature taxon *Akkermansia* in ELE treated mice showed strong positive correlation with TUDCA (*R*^2^ = 0.53).

### Effects of ELE on the TGR5 and Tight Junction Protein Expression in Mice

**FIGURE 5:**
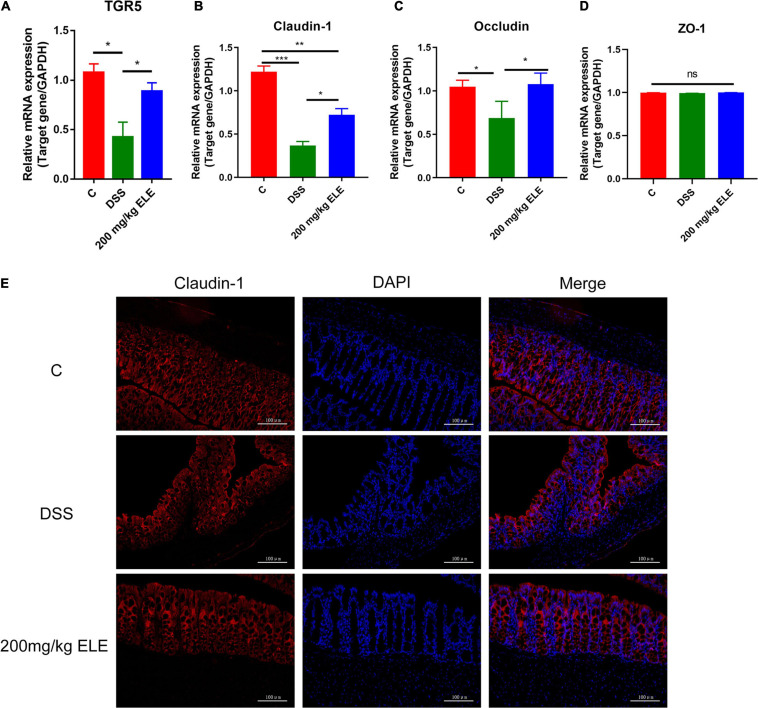
Oral ELE improves DSS-induced decrease of TGR5 and tight junction expression in colon. **(A**–**D)** mRNA expression level of TGR5, claudin-1, occludin, and ZO-1, *n* = 7–8 mice/group. **(E)** Representative image of tissue immunofluorescence of claudin-1, *n* = 3 mice/group. Differences in data in mouse subjects were assessed by one-way ANOVA. *p < 0.05; **p < 0.01; ***p < 0.001.

Based on the change in bile acid content in serum, we further detected the mRNA expression levels of TGR5, a bile acid receptor and tight junction protein in the mouse colons. The data showed that, compared with DSS treatment, ELE treatment significantly increased the decreased mRNA levels of TGR5, claudin-1, and occludin (*p* < 0.05, Figures 5A–C) while had no significant effect on ZO-1 (Figure 5D). Immunofluorescence results showed that claudin-1 was expressed from the top to the bottom of the crypt in the control group. Dextran sulfate sodium administration decreased this expression due to the destruction of intestinal epithelial structure. After ELE treatment, the expression of claudin-1 gradually increased from the bottom to the top of the crypt, suggesting that the expression and distribution of claudin-1 are involved in the process of recovery (Figure 5E).

### TGR5 Involved in the Enhancement of Monolayer Cell Barrier Function

**FIGURE 6:**
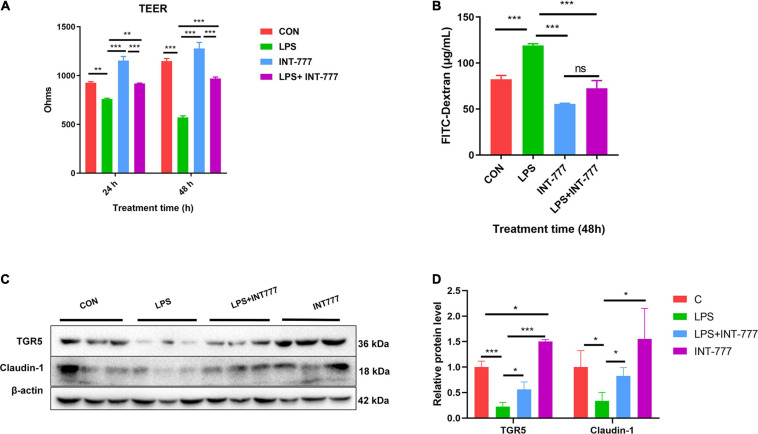
Activation of TGR5 can alleviate LPS-induced epithelium barrier injury of Caco-2 cell. **(A)** The TEER of the cell monolayer of Caco-2, *n* = 3. **(B)** The FITC-dextran permeability of the cell monolayer of Caco-2, *n* = 3. **(C,D)** Protein expression of TGR5 and Claudin-1 relative to β-actin. The results were confirmed by three independent experiments. Differences in data in cell subjects were assessed by one-way ANOVA. *p < 0.05; **p < 0.01; ***p < 0.001.

**FIGURE 7:**
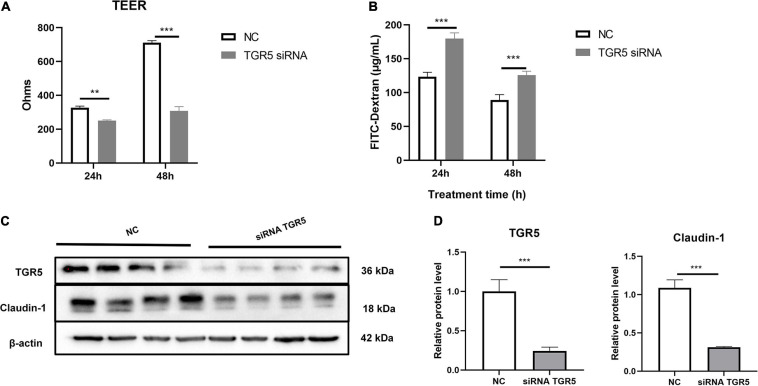
Interference with TGR5 mRNA expression reduces Caco-2 monolayer epithelium barrier function. **(A)** The TEER of the cell monolayer of caco-2, *n* = 3. **(B)** The FITC-dextran permeability of the cell monolayer of Caco-2, *n* = 3. **(C,D)** Protein expression of TGR5 and Claudin-1 relative to β-actin. The results were confirmed by three independent experiments. Differences in data in cell subjects were assessed by one-way ANOVA. *p < 0.05; **p < 0.01; ***p < 0.001.

In this study, we used Caco-2, a human colon epithelium cell line, to confirm the role of bile acid receptor TGR5 in alleviating the process of colon epithelium injury. To activate TGR5, a selective agonist INT-777 was used. The results showed that INT-777 prevented the LPS-induced decrease in the TEER value in the Caco-2 monolayer cell barrier and increase in FITC-dextran permeability (Figures 6A,B). Western blotting data showed that INT-777 significantly increased LPS-induced reductions in claudin-1 protein level (Figures 6C,D), suggesting that INT-777 can promote the barrier function of colon cells. TGR5-siRNA was then used for TGR5 mRNA interference and TEER and FITC-dextran permeability were also evaluated after treatment for 24 and 48 h. The results showed that interference with TGR5 expression could significantly decrease TEER and increase the permeability of FITC-dextran in monolayer cells (*p* < 0.05, Figures 7A,B). Western blotting results showed that, TGR5 interference significantly decreased claudin-1 expression (*p* < 0.05, Figures 7C,D).

## Discussion

To date, there is no ideal drug to treat IBD. Conventional treatment mainly includes oral intake antibacterial and anti-inflammatory drugs, or taking surgery, etc. In recent years, researchers have found that effective components in plant can be used to prevent or treat intestinal inflammation (Zhao et al., 2019; Long et al., 2021). *Eucommia ulmoides* is a traditional Chinese herbal medicine that has also been approved for use as a functional food. *Eucommia ulmoides* has anti-inflammatory and wound-healing promoting effects, but its protective effect on animals has not been fully elucidated. Here, based on our findings, we discuss the effect of ELE on mouse IBD and its potential mechanisms. To better elucidate the mechanisms by which ELE alleviates IBD, we first determined the main components in the extract. The results showed that the main components were phenolic acids, total flavonoids, and sugars. Furthermore, the LC-MS showed chlorogenic acid and geniposide as the most abundant ingredients in ELE. In this study, we constructed a mild enteritis model in mice treated with 2% DSS for 7 days. ELE significantly alleviated the DSS-induced diarrhea rate and colon shortening in mice. Furthermore, we found that ELE significantly improved DSS-induced colon epithelial injury and reduced the mRNA expression of TLR4 and IL-6, which might attribute to the enriched phenolic acids, flavonoid, and polysaccharide contents in ELE. Previously, studies have shown that the phenolic acid, total flavonoids, and polysaccharides alleviate IBD by relieving inflammation and oxidative stress (Zhao et al., 2019; Hussain et al., 2020; Sandoval-Ramirez et al., 2020). Chlorogenic acid, the main component of ELE, showed beneficial effect on ameliorating LPS-induced inflammation in different cell types (i.e., macrophages, hepatocytes, and intestinal cells) by suppressing the TLR4 signaling pathway (Shi et al., 2013; Ruan et al., 2014; Ruifeng et al., 2014). TLR4 is the receptor of lipopolysaccharide, the main component of the cell wall of Gram-negative bacteria. TLR4 can resist the invasion of pathogens such as *Enterobacter* and *Bacteroides* by initiating the inflammatory response of the body (Lu et al., 2018). In this study, the change in TLR4 expression suggests that ELE may promote the recovery of IBD by inhibiting the binding of antigens from the intestinal microbiota. In addition to directly participating in the wound-healing process of the gut epithelium, in recent years, studies have also shown that the “gut microbiota-bile acid metabolism” axis is critical for flavonoids, phenolic acids, and polysaccharides to regulate intestinal health (Zhang et al., 2016; Guo et al., 2019; Huang et al., 2019).

*Bacteroides* have been reported to induce IBD and worsen the disease by producing enterotoxins, degrading mucin, activating TLRs, and inducing the secretion of proinflammatory cytokines (Chung et al., 2018; Elahi, 2018; Menghini et al., 2019). Numerous studies have shown that the abundance of *Bacteroides* is significantly increased in both DSS-induced mice and IBD patients (Rodriguez-Palacios et al., 2018; Wang K. et al., 2018; Ryan et al., 2020). In this study, the reduction in OS, richness, and diversity of gut microbiota were improved by ELE in IBD mice. Furthermore, ELE decreased *Bacteroidaceae* and *Erysipelotrichaceae* abundance and increased *Burkhoderiaceae*, *Akkermansiaceae*, and *Ruminococcaceae* abundance compared with the DSS group, which is in accordance with TLR4 mRNA upregulation. In addition, we found that *Akkermansia* was a feature taxon in ELE-treated mice by LEfSe analysis. Alternatively, *Akkermansiaceae* have been considered probiotics involved in reducing inflammation, promoting intestinal epithelial wound healing, and alleviating enteritis (Zhang et al., 2020). *Akkermansia muciniphila* can significantly alleviate DSS-induced chronic colitis by reducing the expression of TNF-α and IFN-γ, promoting the differentiation of Treg cells, and increasing the production of short-chain fatty acid (Zhai R. et al., 2019). Consistent with a previous study, caffeic acid, which is also a main components and metabolite of chlorogenic acid in ELE, can ameliorate DSS-induced colitis and increase the abundance of *Akkermansia* (Zhang et al., 2016).

Although homeostasis of the gut microbiota is crucial for intestinal health, the specific mechanism has not been fully elucidated. Studies have shown that the intestinal metabolites, which play important roles in host intestinal inflammatory response, cell proliferation, apoptosis and other processes, can be regulated by gut microbiota (Koh and Backhed, 2020).

Bile acid is a type of small molecule metabolite that is abundant in the intestine and is considered a biomarker involved in gut health and the pathogenesis of IBD. As early as in the 1980s, researchers found that there was a high correlation between blood bile acid content and IBD (Holzbach et al., 1980). The type and content of bile acids are highly correlated with the structure of intestinal flora. *Enterobacteriaceae* and *Bacteroides* are highly positively correlated with enteritis, and affected the metabolism of bile acids in the liver and gut. Differences in fecal bile acid and the microbial community were found in the children with remission and non-remission of Crohn’s disease. Primary bile acids (CA, CDCA) were dominant in the non-remission fecal samples of children, and were a highly positively correlated with the higher abundance of *Bacteroidetes*. In contrast, secondary bile acids were dominant in the remission fecal samples of children and were positively correlated with *Rominococcus_torques* belonging to *Firmicutes* (Connors et al., 2020). Similarly, LCA and DCA, but not CDCA, were found to have alleviation effect of DSS- and TNBS-induced colitis (Sinha et al., 2020). Furthermore, 3β-hydroxydeoxycholic acid, a derivate of DCA can act on dendritic cells and promote the differentiation of regulatory T cells, thus alleviating inflammation (Campbell et al., 2020). Alternatively, some researchers found that increased levels of BAs, especially secondary bile acids (DCA and ω-MCA) showed negative correlations with intestinal permeability, and decreased the expression levels of tight junctions (Murakami et al., 2016). These hydrophobic bile acids may induce cytotoxicity, oxidative DNA damage, cell apoptosis, and mitochondrial perturbations in the colon (Barrasa et al., 2013). In this study, oral administration of ELE restored the diversity and abundance of intestinal microbiota in mice with IBD and reduced the abundance of *Bacteroides*. Consistent with the results in this study, we also found that in IBD mice, the level of primary bile acids such as CA, β-MCA, and Tβ-MCA are increased. The level of secondary bile acid (DCA, TDCA) was recovered to a similar level compared with normal mice after ELE treatment. We also found that the level of TUDCA was positively correlated with the abundance of *Akkermansia*. Tauroursodeoxycholic acid is an effective drug that is approved by the US Food and Drug Administration (FDA) for bile acid metabolism regulation, hepatocyte protection, and biliary cholangitis treatment. Tauroursodeoxycholic acid has anti-inflammatory effects in steatohepatitis and non-alcoholic fatty liver disease through alleviation of endoplasmic reticulum stress and downregulation of inflammatory responses (Wang W. et al., 2018). Studies have shown that the mechanism of TUDCA, LCA, and DCA in alleviating enteritis is to activate the bile acid receptor TGR5 in colon (Kusaczuk, 2019; Sorrentino et al., 2020). The bile acid receptor TGR5 plays important roles in the occurrence, development, and recovery of intestinal inflammation. Activation of TGR5 can significantly improve the proliferation of intestinal stem cells and promote intestinal epithelial reconstruction in DSS-induced enteritis mice, but TGR5 knockout can hinder the recovery of the intestinal tract (Sorrentino et al., 2020). Activation of TGR5 not only promotes cell proliferation but also enhances the barrier function of epithelial tissue in mice (Cipriani et al., 2011; Merlen et al., 2020). Consistent with a previous study, we found that compared with DSS treatment, ELE significantly increased the mRNA expression of TGR5 in the colon of IBD mice. To further clarify the role of TGR5 in the regulation of gut epithelial barrier function in the colon, in Caco-2 cell model, the expression of TGR5 was found to be significantly decreased by LPS and RNA interference treatment, while the permeability of TEER and macromolecules (FITC-dextran) permeability was increased. This phenomenon was reversed after the addition of INT-777, a TGR5 selective agonist, indicating that TGR5 is necessary to maintain the barrier function of intestinal epithelial cells. However, in the porcine jejunal epithelial cell line IPEC-J2, mRNA interference with TGR5 did not affect the function of intestinal barrier (Song et al., 2019), which may be a result from the different expression levels of TGR5 in different intestinal sections. TGR5 was found to be highly expressed in the colon and lowly expressed in the jejunum (Wahlstrom et al., 2016); this suggests that TGR5 plays different roles in the gastrointestinal tract. In addition, due to the difference of species and cell types, the function of TGR5 may be different. Therefore, the role of TGR5 on the function of epithelial barrier is worth further study.

In future studies on mice or human subjects, some aspects still need to be clarified, including the effects of long-term use of ELE on intestinal health and gut microbiota regulation and a single compound or multiple compounds within the ELE exert protective effects from IBD and gut barrier injury through the “intestinal microbiota-bile acid-TGR5” axis. In addition, and the potential mechanisms and pathways by which TGR5 regulates the intestinal barrier also need to be further studied.

## Conclusion

In summary, our findings demonstrate that ELE alleviates DSS-induced IBD. Furthermore, we found that ELE treatment significantly increased serum bile acids such as TUDCA and DCA, which showed a high positive correlation with *Akkermansia* and *Rominococcus*. The change of bile acid composition upregulated the expression of TGR5 mRNA in colon tissues. In Caco-2 cells, the activation of TGR5 enhanced cell barrier function and upregulated the expression of tight junction proteins, and this result was reversed by TGR5 mRNA interference. These findings suggest that ELE may be useful as a functional food in alleviating IBD through “gut microbiota-bile acid-TGR5” axis.

## Data Availability Statement

The data that support the findings of this study are openly available in National Center for Biotechnology Information (NCBI), BioProject accession number PRJNA730339.

## Ethics Statement

All the experiment operations were conducted following the guidelines of the institute of Subtropical Agriculture on Animal Care, Chinese Academy of Sciences (No. ISA-2020-18).

## Author Contributions

ZZ, K-MN, and XW conceived and designed the experiments. ZZ, K-MN, YL, and CL performed the experiments and collected the samples. ZZ and K-MN analyzed the data. XW provided the funding. All authors read and approved the final manuscript.

## Conflict of Interest

The authors declare that the research was conducted in the absence of any commercial or financial relationships that could be construed as a potential conflict of interest.

## Publisher’s Note

All claims expressed in this article are solely those of the authors and do not necessarily represent those of their affiliated organizations, or those of the publisher, the editors and the reviewers. Any product that may be evaluated in this article, or claim that may be made by its manufacturer, is not guaranteed or endorsed by the publisher.
