# Supplementary material for: The Gut Microbiota-Bile Acids-TGR5 Axis Mediates Eucommia ulmoides Leaf Extract Alleviation of Injury to Colonic Epithelium Integrity
Source: Front Microbiol. 2021 Aug 18;12:727681. doi: 10.3389/fmicb.2021.727681 (PMC8416499; doi:10.3389/fmicb.2021.727681)
Supplement: Supplementary file 1 [file Data_Sheet_1.doc]

Supporting Information

|  | **Name** | **Formula** | **Molecular Weight** | **RT [min]** | **Peak Area** |
| --- | --- | --- | --- | --- | --- |
| 1 | Chlorogenic acid | C_16_H_18_O_9_ | 354.0965 | 5.106 | 342333012.9 |
| 2 | Geniposidic acid | C_16_H_22_O_10_ | 374.12289 | 3.864 | 298151890.4 |
| 3 | Geniposidic acid | C_16_H_22_O_10_ | 374.1226 | 3.666 | 222138745.9 |
| 4 | L-(-)-Malic acid | C_4_H_6_O_5_ | 134.02108 | 1.369 | 11568046.31 |
| 5 | Quercetin | C_15_H_10_O_7_ | 302.04383 | 8.323 | 10145676.9 |
| 6 | (+)-Catechin hydrate (+)- | C_15_H_14_O_6_ | 290.08021 | 5.647 | 5591802.886 |
| 7 | L-(-)-Malic acid | C_4_H_6_O_5_ | 134.02111 | 1.417 | 4826455.339 |
| 8 | Succinic acid | C_4_H_6_O_4_ | 118.02606 | 1.996 | 3587991.223 |
| 9 | Luteolin | C_15_H_10_O_6_ | 286.04884 | 8.265 | 3068273.805 |
| 10 | Azelaic acid | C_9_H_16_O_4_ | 188.10514 | 7.481 | 2498826.178 |
| 11 | L-Threonic acid | C_4_H_8_O_5_ | 136.03667 | 1.265 | 2307432.85 |
| 12 | (+)-Catechin hydrate (+)- | C_15_H_14_O_6_ | 290.08021 | 5.062 | 2245545.67 |
| 13 | Naringenin | C_15_H_12_O_5_ | 272.06961 | 9.087 | 1909523.026 |
| 14 | Caffeic acid | C_9_H_8_O_4_ | 180.04232 | 5.662 | 1745648.092 |
| 15 | 4-Coumaric acid | C_9_H_8_ O_3_ | 164.04724 | 6.56 | 1553102.886 |
| 16 | Gluconic acid | C_6_H_12_O_7_ | 196.05847 | 1.236 | 1336396.662 |
| 17 | Esculin | C_15_H_16_O_9_ | 340.0807 | 5.373 | 1071836.548 |
| 18 | Naringenin | C_15_H_12_O_5_ | 272.06955 | 7.137 | 639181.7961 |
| 19 | 4-Hydroxybenzoic acid | C_7_H_6_O_3_ | 138.03134 | 8.126 | 626729.0061 |
| 20 | Caffeic acid | C_9_H_8_O_4_ | 180.04237 | 5.212 | 520279.5272 |
| 21 | 3-Coumaric acid | C_9_H_8_O_3_ | 164.04724 | 5.243 | 434777.64 |
| 22 | 2,4-Dihydroxybenzoic acid | C_7_H_6_O_4_ | 154.02643 | 6.119 | 337579.4835 |
| 23 | N-Acetyl-DL-tryptophan | C_13_H_14_N_2_O_3_ | 246.10139 | 6.908 | 257544.7021 |
| 24 | N-Acetyl-L-phenylalanine | C_11_H_13_NO_3_ | 207.08993 | 6.568 | 235766.9175 |
| 25 | 2-Isopropylmalic acid | C_7_H_12_O_5_ | 176.06855 | 5.268 | 226827.2241 |
| 26 | Pantothenic acid | C_9_H_17_NO_5_ | 219.11118 | 3.726 | 171982.1083 |
| 27 | Suberic acid | C_8_H_14_O_4_ | 174.08918 | 6.572 | 164589.0243 |
| 28 | Dehydroacetic acid | C_8_H_8_O_4_ | 168.04239 | 7.33 | 160387.8874 |
| 29 | Caffeic acid | C_9_H_8_O_4_ | 180.04227 | 6.003 | 152461.6521 |
| 30 | Apigenin | C_15_H_10_O_5_ | 270.05392 | 8.986 | 1743798.642 |

Table 1. Top 30 small molecule metabolites in *Eucommia ulmoides* leaves extract

RT: retention time

Table 2. The primer information used in qPCR

|  | Reference sequence | Sense（5‘-3’） | Anti-sense（5‘-3’） | Product length | Tm（℃） |
| --- | --- | --- | --- | --- | --- |
| TGR5 | NM_174985.1 | GCGATGTACCCTCAACCCTG | TTGTCCCTCTTGGCTCTTCC | 307 | 60 |
| TLR4 | NM_021297.2 | GGAACAAACAGCCTGAGACACTT | CAAGGGATAAGAACGCTGAGAA | 151 | 60 |
| IL-6 | NM_031168.2 | CCCCAATTTCCAATGCTCTCC | CGCACTAGGTTTGCCGAGTA | 141 | 60 |
| IL-10 | NM_010548.2 | TTTAAGGGTTACTTGGGTTGCC | AATGCTCCTTGATTTCTGGGC | 106 | 60 |
| ZO-1 | NM_009386.2 | GGGAAAACCCGAAACTGATG | GCTGTACTGTGAGGGCAACG | 103 | 60 |
| Claudin-1 | NM_016674.4 | ATTTCAGGTCTGGCGACATTAGT | TGTTGGGTAAGAGGTTGTTTTCC | 204 | 60 |
| Occludin | [NM_001360538.1](https://www.ncbi.nlm.nih.gov/entrez/viewer.fcgi?db=nucleotide&id=1339869085) | ATGTCCGGCCGATGCTCTC | TTTGGCTGCTCTTGGGTCTGTAT | 308 | 60 |
| GAPDH | NM_008084.2 | CCTCGTCCCGTAGACAAAATG | TGAGGTCAATGAAGGGGTCGT | 133 | 60 |

Table 3 The standard curve of 25 BAs

| Component | RT (min) | Equation | R |
| --- | --- | --- | --- |
| LCA | 11.14 | y = 0.01060 x + 0.03837 | 0.99921 |
| 7-KLCA | 9.37 | y = 0.00380 x + 0.00113 | 0.9971 |
| 12-KLVA | 9.57 | y = 0.01478 x + 1.07696e-5 | 0.9996 |
| UDCA | 8.6 | y = 0.00732 x + 2.62687e-4 | 0.99709 |
| CDCA | 10.05 | y = 0.00411 x + 0.00545 | 0.99559 |
| DCA | 10.19 | y = 0.00959 x + 0.01060 | 0.99775 |
| ω-MCA | 4.94 | y = 0.00624 x - 0.00145 | 0.9996 |
| α-MCA | 5.41 | y = 0.00333 x - 1.75774e-5 | 0.99949 |
| β-MCA | 6.01 | y = 0.00522 x - 0.00134 | 0.99804 |
| HCA | 7.16 | y = 0.00376 x - 0.00120 | 0.99958 |
| CA | 8.21 | y = 0.02926 x + 0.00458 | 0.99863 |
| GLCA | 9.37 | y = 0.01122 x + 0.00222 | 0.99882 |
| GUDCA | 3.07 | y = 0.02132 x - 0.00240 | 0.99927 |
| GCDCA | 5.67 | y = 0.01167 x + 0.00268 | 0.99919 |
| GDCA | 6.39 | y = 0.01001 x - 0.00172 | 0.9993 |
| GDHCA | 1.99 | y = 0.00474 x + 6.56393e-5 | 0.99885 |
| GHCA | 2.46 | y = 0.05285 x + 0.01037 | 0.9931 |
| GCA | 3.13 | y = 0.11440 x + 0.00287 | 0.99898 |
| TLCA | 8.53 | y = 0.22268 x + 0.04316 | 0.99784 |
| TUDCA | 2.64 | y = 0.02141 x + 0.00115 | 0.99947 |
| TCDCA | 4.74 | y = 0.00970 x - 0.00137 | 0.99994 |
| TDCA | 5.4 | y = 0.01197 x - 0.00807 | 0.99361 |
| TDHCA | 1.84 | y = 0.00524 x - 6.12343e-4 | 0.99885 |
| Tβ-MCA | 1.86 | y = 0.02459 x - 0.00258 | 0.99822 |
| TCA | 2.78 | y = 0.01180 x - 6.30254e-4 | 0.99994 |

Table 4 The internal standard used in BA anylasis

|  | CAS | Name | Isotope labled |
| --- | --- | --- | --- |
| 1 | 116380-66-6 | Cholic acid-d4 | Yes |
| 2 | 99102-69-9 | Chenodeoxycholic acid-d4 | Yes |
| 3 | 112076-61-6 | Deoxycholic acid-d4 | Yes |
| 4 | 1201918-16-2 | Glycochenodeoxycholic acid-d4 | Yes |
| 5 | 1069132-37-1 | Glycodeoxycholic acid-d4 | Yes |
| 6 | 83701-16-0 | Glycolithocholic acid-d4 | Yes |
| 7 |  | Lithocholic acid-d4 | Yes |
| 8 | 252030-90-3 | Taurocholic acid-d4 | Yes |
| 9 |  | Tauroursodeoxycholic acid-d5 | Yes |
| 10 |  | 2-Chloro-L-phenylalanine | No |

Table 5. The chromatographic elution gradient

| Time (min) | aqueous phase (%) | organic phase (%) |
| --- | --- | --- |
| 0.00 | 95.00 | 5.00 |
| 0.50 | 60.00 | 40.00 |
| 4.50 | 50.00 | 50.00 |
| 7.50 | 25.00 | 75.00 |
| 10.00 | 5.00 | 95.00 |
| 12.00 | 95.00 | 5.00 |

Table 6. The siRNA sequence of TGR5

|  | Sense | Antisense |
| --- | --- | --- |
| NC | UUCUCCGAACGUGUCACGUTT | ACGUGACACGUUCGGAGAATT |
| TGR5-001 | UCGACCUGGACUUGAACUAAATT | UUUAGUUCAAGUCCAGGUCGATT |

NC: negative control sequence

Supporting information Figure 1


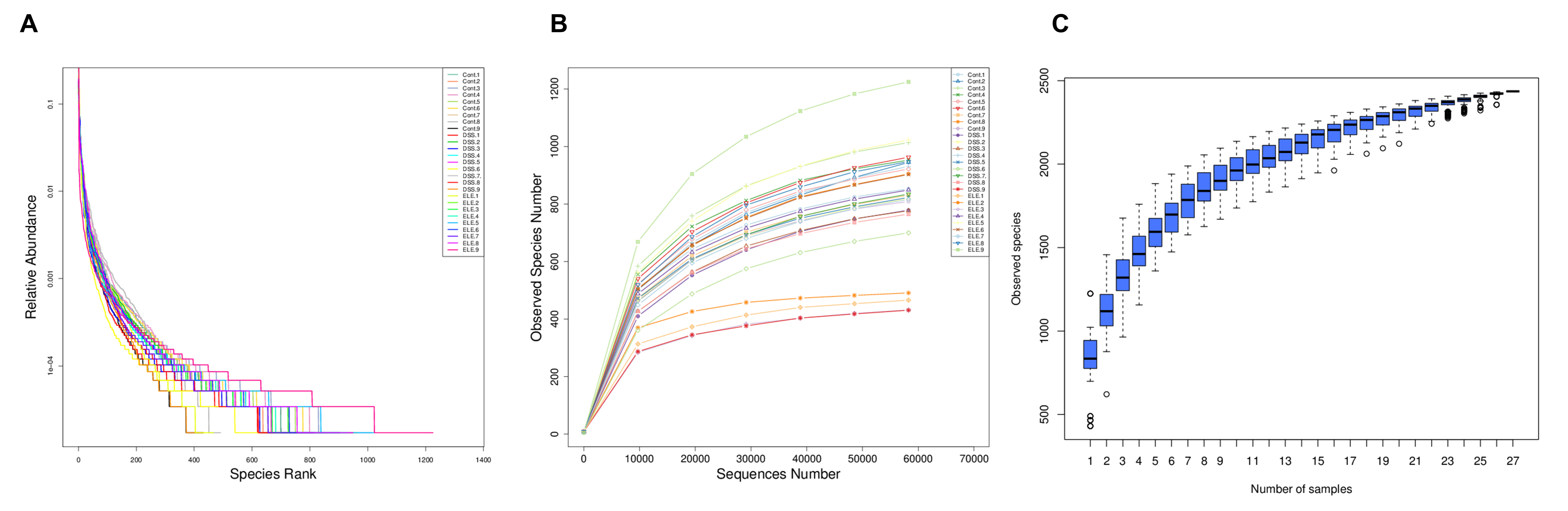


**Figure S1.** The rank abundance curve (A), Rarefaction curve (B) and species accumulation boxplot (C).
